# Anemia and Feeding Practices among Infants in Rural Shaanxi Province in China

**DOI:** 10.3390/nu6125975

**Published:** 2014-12-22

**Authors:** Renfu Luo, Yaojiang Shi, Huan Zhou, Ai Yue, Linxiu Zhang, Sean Sylvia, Alexis Medina, Scott Rozelle

**Affiliations:** 1 Center for Chinese Agricultural Policy, Institute of Geographical Sciences and Natural Resources Research, Chinese Academy of Sciences, No. Jia 11, Datun Road, Chaoyang, Beijing 100101, China; E-Mails: luorf.ccap@igsnrr.ac.cn (R.L.); lxzhang.ccap@igsnrr.ac.cn (L.Z.); 2 Center for Experimental Economics in Education (CEEE), Shaanxi Normal University, 620 Chang’an Rd West, Xi’an 710119, China; E-Mails: shiyaojiang7@gmail.com (Y.S.); yueai@163.com (A.Y.); 3 West China School of Public Health, Sichuan University, No. 17, Section 3, South Renmin Road, Chengdu 610041, China; 4 School of Economics, Renmin University of China, 59 Zhongguancun Avenue, Beijing 100872, China; E-Mail: ssylvia@stanford.edu; 5 Freeman Spogli Institute for International Studies, Stanford University, 616 Serra Street, Stanford, CA 94305, USA; E-Mails: amedina5@stanford.edu (A.M.); rozelle@stanford.edu (S.R.)

**Keywords:** anemia, nutrition, feeding practices, infant, rural China

## Abstract

Anemia is one of the most prevalent public health problems among infants and iron deficiency anemia has been related to many adverse consequences. The overall goal of this study is to examine the prevalence of anemia among infants in poor rural China and to identify correlates of anemia. In April 2013, we randomly sampled 948 infants aged 6–11 months living in 351 villages across 174 townships in nationally-designated poverty counties in rural areas of southern Shaanxi Province, China. Infants were administered a finger prick blood test for hemoglobin (Hb). Anthropometric measurement and household survey of demographic characteristics and feeding practices were conducted in the survey. We found that 54.3% of 6–11 month old infants in poor rural China are anemic, and 24.3% of sample infants suffer from moderate or severe anemia. We find that children still breastfed over 6 months of age had lower Hb concentrations and higher anemia prevalence than their non-breastfeeding counterparts (*p* < 0.01), and that children who had ever been formula-fed had significantly higher Hb concentrations and lower anemia prevalence than their non-formula-fed counterparts (*p* < 0.01). The results suggest the importance of iron supplementation or home fortification while breastfeeding.

## 1. Introduction

Anemia is one of the most prevalent nutritional disorders, especially among preschool-aged children in developing countries [1]. Although anemia is broadly influenced by a variety of factors including nutrition, infectious disease, and genetics, iron deficiency is the major cause of anemia, accounting for about half of the global incidence of anemia [2,3,4]. In China, it is postulated that 90% of anemia in infants and young children results from iron deficiency [5].

Iron deficiency anemia in the first years of life has been related to many adverse consequences. In the short term, infants with iron deficiency anemia are at higher risk of cognitive, social and emotional delays [6,7,8,9,10,11,12,13,14]. In the longer run, it can negatively affect school performance and behavior, reduce overall educational attainment, and negatively affect work outcomes into adulthood; these consequences are irreversible even if the anemia is corrected in later childhood [15,16,17,18]. Many of these consequences can be avoided, however, simply through proper dietary intake in the first years of life; studies have shown that regular iron supplementation can reduce anemia rates by as much as 50% [19].

Despite overall improvements in child health in China in recent decades, between 1992 and 2005 there was no significant decrease in the prevalence of anemia among children under 5 years old [20,21,22,23]. Recent studies have found consistently high rates of anemia among this population, even as standards of living improved. The prevalence of anemia among infants aged 6–12 months living in rural areas was 35% in 2005, while a national survey of both urban and rural children found the average anemia prevalence of 6–12 month olds to be 28.2% [23,24]. Other, more geographically focused studies have found similarly high rates, ranging from 23% (in Guangxi) [25] to 58% (in Gansu) [26].

Although there have been a number of studies measuring the prevalence of anemia among infants and young children in China, most of these studies have been based on relatively small sample sizes [25,27]. Further, few studies have sought to thoroughly assess correlates and risk factors of anemia in this population [28,29]. The correlational studies that do exist only consider a narrow set of potential correlates. Some studies, for instance, focused only on geographical location, comparing differences in infant anemia prevalence between rural and urban China while others have only considered the bivariate associations between anemia and basic infant and household characteristics [23,25,26,27,28,30,31,32]. Examining a richer set of correlates and risk factors is essential to painting a more complete picture of the population most at risk.

One factor of particular interest in this context is feeding practices. The relationship between feeding practices and anemia is of considerable policy relevance since feeding behaviors can—in theory—be affected through policy interventions more easily than can more permanent socioeconomic characteristics such as educational attainment or household income. Despite this, however, there is only a handful of large-scale studies that consider the relationship between infant anemia and feeding practices in rural China, with inconclusive results [26,27,29,32]. Only one of these studies used multiple regression analysis to control for potential confounding factors.

The overall goal of this study is to examine the prevalence of anemia among infants in China’s poor rural areas and to identify correlates of anemia. We find 54.3% anemia prevalence among the infants in our sample, and that children weaned after 6 months have significantly lower rates of anemia relative to children who are still exclusively or predominantly breastfed after 6 months.

## 2. Materials and Methods

### 2.1. Sample Selection

In 2013, we conducted a cluster-randomized cross-sectional study of anemia prevalence among infants aged 6–11 months in 11 nationally-designated poverty counties located in southern Shaanxi province in northwestern China. In each of these 11 counties, all townships (the middle level of administration between county and village) were selected to participate in the study. We excluded the township in each county that housed the county seat. In total, the sample included 174 townships in 11 sample counties.

We randomly selected one village from each sample township to participate in the study. A list of all registered births over the past 12 months was obtained from the local family planning official in each village. All babies in the sample village in our target age range (6–11 months) were enrolled in the study. To meet the power requirements of a larger, interventional study (not reported in this paper), we required a minimum of five babies in each township. With this requirement in mind, if a village had fewer than five eligible infants, we randomly selected an additional village in the same township for inclusion in the study. All eligible infants in this second village were also enrolled in the study. Overall then, our study included 948 infants in 351 villages across 174 townships. The survey was carried out over a 20 day period in April 2013.

### 2.2. Data Collection

With the assistance of trained nurses from Xi’an Jiaotong Medical School, we collected hemoglobin (Hb) concentrations from all participating infants. In order to measure Hb concentrations in sample infants, a single drop of capillary blood was obtained with pressure activated safety lancets and analyzed using a HemoCue Hb 201+ system (Hemocue, Inc, Ängelholm, Sweden). The HemoCue Hb 201+ system was selected for the study because it was fast, accurate, and convenient and was suitable for use in outpatient units [33]. The nurses also measured the length of each infant to a precision of 0.1 cm and the weight of each infant to a precision of 0.1 kg, following the procedural guidelines recommended by the World Health Organization (WHO) [34].

Teams of trained enumerators collected socioeconomic and demographic data from all households participating in the study. During the interview, each infant’s primary caregiver was identified. The primary caregiver was identified in each sample household by asking which family member was most responsible for the infant’s care. (In over 98% of the cases, the primary caregiver was either the infant’s mother or paternal grandmother.) Enumerators administered a detailed survey on parental and household characteristics, including each infant’s gender and birth order. We also collected information on the mother’s age and level of education. Each family also reported whether they received Social Security Support (payments from China’s Minimum Living Standard Guarantee system), which we use as a household-specific indicator of poverty. China’s social security payments have been shown to be well-targeted and reliable indicators of poverty in China [35]. Each infant’s age was obtained from his or her birth certificate.

The survey also included a detailed module on feeding practices, based on the “Indicators for assessing infant and young child feeding practices” [36,37] compiled by UNICEF, USAID, the World Health Organization, and others in the international community. Careful month-by-month histories of breastfeeding and formula feeding were taken from each household. Enumerators also documented the introduction of solid foods into the infant’s diet. Breastfeeding is defined to include both exclusive and predominant breastfeeding, per WHO definitions [38].

The survey also collected infant health information from the caregiver; specifically, it asked whether the infant had had a fever or diarrhea at any time over the previous month.

### 2.3. Ethical Approval

This study received ethical approval from the Stanford University Institutional Review Board (IRB) (Protocol ID 25734), and from the Sichuan University Ethical Review Board (Protocol ID 2013005-01). All participating caregivers gave their oral consent for both their own and their infant’s involvement in the study. Children who were found to have severe anemia were referred to the local hospital for treatment.

### 2.4. Statistical Analysis

Anemia status was determined based on finger blood analysis for Hb concentrations. Since all of our sample villages were located below 1000 m, the Hb data did not require altitude adjustments. Following internationally accepted standards for our sample age group, anemia was defined as Hb < 110 g/L. We also analyzed the data by sub-category of the sample infants who were suffering from anemia. Mild anemia was defined as 100 g/L < Hb ≤ 110 g/L. Moderate anemia was defined as 70 g/L ≤ Hb < 100 g/L. Severe anemia was defined as Hb < 70 g/L [39,40].

Physical indicators of length and weight were compared with the 2006 WHO child growth standards [41] to calculate length-for-age, weight-for-age, and weight-for-length Z-scores (LAZ, WAZ, WLZ). We followed internationally recognized cutoffs [42] to consider children whose LAZ, WAZ, or WLZ fell more than two standard deviations below the international mean to be stunted, underweight, or wasted, respectively.

Low birth weight was defined to be birth weights below 2500 g.

All statistical analyses were performed using STATA 12.0 (StataCorp, College Station, TX, USA). Statistical significance of bivariate differences in Hb concentrations and anemia prevalence were assessed using ANOVA. Multiple linear regression and logistic regression models were both used in the multivariate analysis.

We considered the following variables as potential confounders in the multivariate analysis: gender, age, birth weight, birth order, whether the infant’s mother was identified as the primary caregiver, maternal educational level and age, whether the infant’s family received Social Security Support, and whether the infant had fever or diarrhea last month. We also clustered our standard errors at the village level to control for any possible intra-village correlation.

## 3. Results

The basic socioeconomic and demographic characteristics of study participants are reported in Table 1. Of the 948 infants in this study, slightly over half (52.6%) of the sample infants were male. Around 5.0% of the sample infants were low birth weight; 61.4% were first-order births. The mother was the primary caregiver for 79% of the 6–11 month old infants in the sample. The majority of the mothers (84.2%) had completed 9 years of schooling or less; 52.5% were over 25 years of age. About one-quarter (24.2%) of sample families reported receiving Social Security Support and can be classified as “poor”.

**Table 1:** Basic characteristics of sample infants in rural Shaanxi Province (*N* = 948).

Characteristics	Frequency (*n*)	Percentage (%)
Gender		
Male	499	52.6
Female	449	47.4
Infant Age		
6 Months	137	14.5
7 Months	183	19.3
8 Months	162	17.1
9 Months	127	13.4
10 Months	173	18.2
11 Months	166	17.5
Low Birth Weight?		
No	901	95.0
Yes	47	5.0
Birth Order of Infant		
First	582	61.4
Second or Higher	366	38.6
Mother is Primary Caregiver		
No	199	21.0
Yes	749	79.0
Years of Maternal Education		
≤9 years	798	84.2
>9 years	150	15.8
Maternal Age		
Age ≤ 25	450	47.5
Age > 25	498	52.5
Families Receive Social Security Support		
No	718	75.8
Yes	230	24.2

Data are presented as frequency and percent for all infants.

### 3.1. Hb Concentration, Anemia and Growth Status of Infants in Rural China

Table 2 summarizes the mean Hb concentrations and anemia prevalence of the sample infants. The mean Hb concentration (g/L) of the sample infants was 107.3 ± 13.0. In our sample, 515 of the 948 infants had Hb concentrations below 110 g/L, resulting in an overall anemia prevalence of 54.3%. The prevalence of mild anemia (100 g/L ≤ Hb < 110 g/L) was 30.0%; the prevalence of moderate anemia (70 g/L ≤ Hb < 100 g/L) was 23.5%; and the prevalence of severe anemia (Hb < 70 g/L) was less than 1%. There was considerable variation in anemia prevalence (Hb < 110 g/L) across the sample counties, ranging from 37.1% to 73.9%.

**Table 2:** Hemoglobin concentration, anemia prevalence, and physical development of sample infants in rural Shaanxi Province (*N* = 948).

Infant Characteristics	Mean/Percent ^a^	95% CI
Hb concentration, g/L	107.3 ± 13.0	106.5–108.2
Anemia status		
Total percent anemic (Hb < 110 g/L)	54.3 (515)	51.1–57.5
Severe anemia (Hb < 70 g/L)	0.8 (8)	0.3–1.4
Moderate anemia (70 g/L ≤ Hb < 100 g/L)	23.5 (223)	20.8–26.2
Mild anemia (100 g/L ≤ Hb < 110 g/L)	30.0 (284)	27.0–32.9
Stunting (LAZ < −2)	3.4 (32)	2.2–4.5
Underweight (WAZ < −2)	1.2 (11)	0.5–1.9
Wasting (WLZ < −2)	1.9 (18)	1.0–2.8

^a^ Data are presented as mean ± SD or % (*n*) for categorical variables. CI, confidence interval.

Table 2 further shows infants’ physical development indicators: length-for-age, weight-for-age, and weight-for-length *Z*-scores. According to our data, 3.4% of infants in our sample were stunted (according to LAZ measures); 1.2% of infants were underweight (according to WAZ measures); and 1.9% of infants were wasted (according to WLZ measures).

### 3.2. Hb Concentrations, Anemia Prevalence and Socioeconomic and Demographic Factors

**Figure 1:**
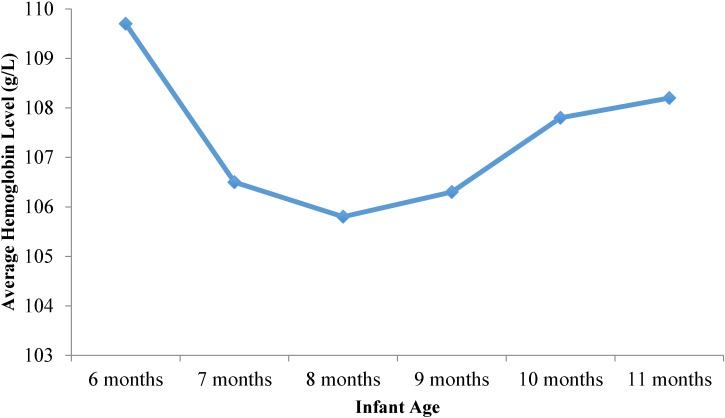
Hemoglobin levels in infants aged 6–11 months by age group in rural Shaanxi province, China, 2013.

We found a U-shaped relationship between infant age and Hb concentrations (Figure 1). Mean Hb concentrations were highest among 6 month old infants (109.7 ± 12.7 g/L), reached a low of 105.8 ± 13.6 g/L among 8 month old infants, and then seemed to recover slightly, reaching 108.2 ± 13.1 g/L among 11 month old infants. The data for anemia prevalence mirror this trend (not shown).

Table 3 presents the bivariate associations between Hb concentrations, anemia prevalence, and infant and household characteristics. There was no difference in Hb concentrations or anemia prevalence either by gender or between lower birth weight and normal birth weight infants. The birth order of infants was not associated with Hb concentration but was significantly correlated with anemia prevalence. When the infant’s mother was identified as the primary caregiver, Hb concentrations were lower and anemia prevalence was higher (*p* < 0.05). Hb concentrations were higher and anemia prevalence was lower among infants whose mothers had 10 or more years of schooling (*p* < 0.01). However, neither mothers’ age nor household economic status (as measured by whether or not the family received Social Security Support) was associated with Hb concentrations or anemia prevalence.

**Table 3:** Hemoglobin concentrations and anemia prevalence of sample infants in rural Shaanxi Province by infant characteristics.

Infant and Household Characteristics	Hb Concentrations (g/L)		Anemic	
	Mean ± SD	*p* Value	% (*n*)	*p* Value
Gender				
Male	107.0 ± 13.6	0.46	55.4 (277)	0.48
Female	107.7 ± 12.2		53.1 (238)	
Low Birth Weight?				
No	107.3 ± 12.8	0.69	54.7 (493)	0.29
Yes	108.1 ± 15.9		46.8 (22)	
Birth Order of Infant				
First	107.9 ± 12.7	0.12	50.9 (296)	<0.01
Second or Higher	106.5 ± 13.3		59.7 (219)	
The First Care-Giver is Mother				
No	109.2 ± 11.6	0.02	46.7 (93)	0.02
Yes	106.8 ± 13.3		56.3 (422)	
Years of Maternal Education				
≤9 Years	106.7 ± 13.0	<0.01	57.0 (455)	<0.01
>9 Years	110.9 ± 12.1		40.0 (60)	
Maternal Age				
Age ≤ 25	106.6 ± 12.8	0.10	55.2 (249)	0.60
Age > 25	108.0 ± 13.1		266(53.5)	
Families Receiving Social Security Support				
No	107.1 ± 13.2	0.26	53.8 (386)	0.54
Yes	108.2 ± 12.0		56.1 (129)	

Data are presented as mean ± SD or % (*n*) for categorical variables.

### 3.3. Hb Concentration, Anemia Prevalence and Infant Feeding Behavior

Table 4 presents the bivariate associations between Hb concentrations, anemia prevalence, and infant feeding behaviors. Infants never breastfed had higher Hb levels and lower rates of anemia than did infants who had ever breastfed (*p* < 0.01). Infants who were exclusively or predominantly breastfed up to 6 months of age and infants who were still breastfed over 6 months of age both had significantly lower Hb levels and higher rates of anemia than their non-breastfeeding counterparts (*p* < 0.01 for all comparisons). By contrast, infants who were ever formula-fed had significantly higher Hb levels and lower rates of anemia than their non-formula feeding counterparts (*p* < 0.01).

There were no statistically significant differences in either Hb concentrations or anemia prevalence between infants first introduced to solid foods before 9 months of age and those first introduced to solids after 9 months of age (Table 4). This may be because most of the solid foods that infants were fed were staples such as porridge and steamed buns. A full 78.3% of infants were fed staples in the 24 h prior to survey administration, but only 23.7% were fed meat in the seven days prior to survey administration (Table 5).

**Table 4:** Hemoglobin concentrations and anemia prevalence of sample infants in rural Shaanxi Province by infant feeding practice (*N* = 948).

Infant Feeding Practice	%	Hb Concentrations (g/L)		Anemic	
		Mean ± SD	*p* Value	% (*n*)	*p* Value
Ever Breastfed					
No	11.8	112.3 ± 12.8	<0.01	37.5 (42)	<0.01
Yes	88.2	106.7 ± 12.9		56.5 (473)	
Exclusive or Predominant Breastfeeding < 6 Months					
No	42.6	110.5 ± 12.4	<0.01	44.9 (182)	<0.01
Yes	57.4	105.0 ± 12.9		61.2 (333)	
Still Breastfed ≥ 6 Months					
No	24.7	112.4 ± 12.2	<0.01	38.9 (91)	<0.01
Yes	75.3	105.7 ± 12.8		59.3 (424)	
Ever Formula-Fed					
No	37.2	103.7 ± 13.0	<0.01	66.2 (234)	<0.01
Yes	62.8	109.5 ± 12.5		47.2 (281)	
Time of Introduction of Solid, Semi-Solid and Soft Food ^a^					
<6 Months	34.5	108.3 ± 12.4	0.09	52.0 (170)	0.31
≥6 Months	65.5	106.8 ± 13.2		55.5 (345)	

Data are presented as mean ± SD or % (*n*) for categorical variables. ^a^ To test the robustness of our analysis, we also ran the analysis on the effect of solid feeding behavior on Hb concentrations and anemia status. Instead of breaking the sample into three categories (less than six months; between six and eight months; greater than eight months) as we did in the manuscript proper, we also broke it into two categories (less than six months; greater than six months). The results (which are not shown for brevity) were substantially the same.

**Table 5:** Feeding practices of sample infants in rural Shaanxi Province (*N* = 948).

Feeding Behavior	Frequency (*n*)	Percentage (%)
Fed staple yesterday		
No	206	21.7
Yes	742	78.3
Fed meat last week		
No	723	76.3
Yes	225	23.7
Fed vegetables last week		
No	430	45.4
Yes	518	54.6
Fed fruits last week		
No	367	38.7
Yes	581	61.3

Data are presented as frequency and percent for all infants.

To better consider the linkages between breastfeeding, introduction of solids, and hemoglobin levels/anemia prevalence, we narrowed our sample to only those children who had never been formula-fed, and considered the relationship between the age at which they were first introduced to solids and both hemoglobin levels and anemia prevalence (Table 6). We found a significant negative relationship between the age at which solids were introduced and hemoglobin levels; children introduced to solids under age 6 months had the highest hemoglobin levels of all non-formula-fed children. There was no significant relationship between the age of introduction of solids and anemia prevalence.

**Table 6:** Hemoglobin concentrations and anemia prevalence among non-formula fed infants in rural Shaanxi Province, by infant feeding practice (*N* = 353).

Time of Introduction of Solid, Semi-Solid and Soft food ^a^	%	Hb concentrations (g/L)		Anemic	
		Mean ± SD	*p* Value	% (*n*)	*p* Value
<6 months	37.1	105.9 ± 12.4	0.02	63.4 (83)	0.39
≥6 months	62.9	102.5 ± 13.2		67.9 (151)	

Data are presented as mean ± SD or % (*n*) for categorical variables; ^a^ To test the robustness of our analysis, we also ran the analysis on the effect of solid feeding behavior on Hb concentrations and anemia status. Instead of breaking the sample into three categories (less than six months; between six and eight months; greater than eight months) as we did in the manuscript proper, we also broke it into two categories (less than six months; greater than six months). The results (which are not shown for brevity) were substantially the same.

### 3.4. Hb Concentration, Anemia Prevalence and Infant Health

Table 7 analyzes the bivariate correlation between Hb concentrations, anemia prevalence and infant health. There was no significant relationship between anemia prevalence and incidence of diarrhea. In contrast, there was a significant association between anemia prevalence and incidence of fever (*p* = 0.01). Rates of anemia were higher among those infants who had experienced fevers than among infants who had not experienced fevers.

**Table 7:** Hemoglobin concentrations and anemia prevalence of sample infants in rural Shaanxi Province by disease characteristics.

Infant Disease Characteristics	*N*	Hb Concentrations (g/L)		Anemic	
		Mean ± SD	*p* Value	% (*n*)	*p* Value
Had fever last month					
No	642	107.9 ± 13.2	0.07	51.6 (331)	0.01
Yes	306	106.2 ± 12.5		60.1 (184)	
Had diarrhea last month					
No	621	107.3 ± 13.0	0.98	54.3 (337)	0.96
Yes	327	107.3 ± 13.0		54.4 (178)	

Data are presented as mean ± SD or % (*n*) for categorical variables.

### 3.5. Multivariate Analysis

Table 8 shows the association between Hb concentrations/anemia prevalence and infant feeding practices after adjusting for potential confounders. We find that children still breastfed over 6 months of age had lower Hb concentrations and higher anemia prevalence than their non-breastfeeding counterparts (*p* < 0.01), and that children who had ever been formula-fed had significantly higher Hb concentrations and lower anemia prevalence than their non-formula-fed counterparts (*p* < 0.01). In contrast to the negative and significant relationship between solid feeding practice and Hb levels found in the univariabe analysis (reported in Table 6), we found no differences in either Hb concentrations or anemia prevalence by age at which solids were first introduced.

**Table 8:** Adjusted association of infant feeding practice, health and nutritional status in rural Shaanxi Province (*n* = 948).

Infant Feeding Practice	Hb Concentrations (g/L) ^a^			Hb < 110 g/L (Yes = 1) ^b^		
	Coefficient	95% CI		Coefficient	95% CI	
Ever breastfed	−0.71	−2.77	1.35	0.01	−0.07	0.09
Exclusive or predominant breastfeeding < 6 months	−0.81	−3.87	2.24	0.05	−0.07	0.18
Still breastfed ≥ 6 months	−5.13 **	−7.60	−2.66	0.13 **	0.03	0.22
Ever formula-fed	3.25 **	1.11	5.39	−0.12 **	−0.20	−0.03
Introduction of solid, semi-solid and soft food ≥ 6 months ^c^	−0.31	−1.88	1.27	0.01	−0.06	0.07

^a^ Regression estimates from multiple linear models adjusted for gender, age, low birth weight, birth order, maternal age, maternal education, whether primary caregiver is mother, whether household received Social Security Support, whether infant had fever or diarrhea last month and county fixed effect. Clustering is at the village level. ^b^ Regression estimates from logit models adjusted for gender, age, low birth weight, birth order, maternal age, maternal education, whether primary caregiver is mother, whether the family received Social Security Support, whether infant had fever or diarrhea last month and county fixed effect. Clustering is at the village level. ^c^ To test the robustness of our analysis, we also ran the analysis on the effect of solid feeding behavior on Hb concentrations and anemia status. Instead of breaking the sample into three categories (less than six months; between six and eight months; greater than eight months) as we did in the manuscript proper, we also broke it into two categories (less than six months; greater than six months). The results (which are not shown for brevity) were substantially the same. ** *p* < 0.01 and * *p* < 0.05.

## 4. Discussion

This study showed that 54.3% of 6–11 month old infants in low-income areas of rural China are anemic. This is about twice as high as the 2011 average for East and Southeast Asia [43]. Over 20% of sample infants suffered from moderate or severe anemia. These results are consistent with results of other studies of anemia among infants in poor rural China [26,27,28,31,32]. The anemia prevalence that we identify in this study classifies anemia in our sample areas as a “severe public health problem” according to the WHO [44]. The WHO further recommends that in communities where iron deficiency anemia prevalence exceeds 40%, all infants and toddlers should receive iron supplementation [44]. This supplementation can take the form of traditional syrups, or home fortification powders.

While it is beyond the scope of this paper to precisely identify the exact source of the high anemia prevalence we find in this study, one possible reason may be that infants in rural China are born with suboptimal iron stores [45]. It is known that iron needs during the first six months of life are primarily supplied by iron stores at birth; infants in well-nourished populations born at term and of normal birth weight typically have sufficient iron stores at birth to prevent iron deficiency until six months of age [46]. The fact that our data show 50.7% anemia prevalence at age 6 months may therefore be indicative of poor maternal iron status during pregnancy. Given that 39% of our sample children were formula fed prior to 6 months of age (data not shown), and therefore would have been receiving additional iron supplementation, this high anemia prevalence at 6 months is even more troubling.

China’s infant nutrition problems appear to stem from poor quality weaning diets rather than insufficient energy intake. Child growth faltering, as measured by stunting, underweight and wasting, are at extremely low levels in the study population, ranging from only 1.2% to 3.4%. Such low rates are indicative of a population with virtually no linear growth failure. This contrast between the lack of linear growth failure and high anemia prevalence suggests that it is the quality of food, rather than the quantity, that is lacking in poor rural China today. This assertion is supported by our data on infant feeding practices, which show that while children seem to be introduced to complementary foods according to a timeline that fits with international guidelines, the typical weaning diet seems to be comprised primarily of staples, with less than one-quarter of sample children eating meat in the seven days prior to survey administration.

We were a bit surprised to see that there was no difference in anemia rates or Hb levels between low birth weight and normal birth weight infants, as the literature suggests otherwise [47,48]. We suspect that the absence of significant difference in our sample may stem from our small sample of low birth weight infants, accounting for only 5.0% of the total sample.

Our results also show that breastfeeding beyond 6 months of age is associated with significantly higher rates of anemia among sample children, although it is important to note that the quality of the weaning diet among the sample population was generally poor. These findings are similar to studies in Mexico and the U.S. in which exclusive breastfeeding for 6 months was associated with an increased risk of infant anemia (compared with exclusive breastfeeding for less than 6 months) [46,49]. Other studies have also identified weaning after 6 months as a risk factor for iron deficiency anemia in resource-limited countries, where infants are more likely to have lower iron stores at birth [50,51,52].

The lower risk of anemia and higher Hb concentrations associated with formula feeding is perhaps not surprising as most infant formulas are fortified with iron and other micronutrients. The negative association between formula feeding and anemia has been reported in several other studies [53,54].

Our findings should not imply any deviation from WHO breastfeeding recommendations. It is well known that there are many benefits to breastfeeding, including protection against infectious morbidity, lower mortality, and promotion of adequate growth and development [38,55]. As a live substance, breast milk is uniquely suited to infants’ nutritional needs, and contains unparalleled immunological and anti-inflammatory properties that protect against a host of illnesses and diseases for both mothers and children. Formula feeding and early weaning from breast milk are linked to higher rates of a number of serious health conditions, ranging from ear infections and diarrhea to leukemia and sudden infant death syndrome [56]. Our results may suggest, however, the importance of either iron supplementation or home fortification systems (possibly including other micronutrients) for breastfeeding infants in developing settings. At the very least, the quality of the weaning diet should be improved to include more and better sources of dietary iron. More study on this important issue is needed.

We have devoted some thought to potentially confounding exogenous factors that may have influenced our study results. One possibility may be that there are seasonal or temporal factors affecting our data that we may have overlooked. Based on previous studies of anemia among school-aged children in sample areas very similar to the ones used in this study, we know that anemia rates tend to be slightly lower in the spring than in the fall [57,58], likely due to the Chinese Spring Festival holiday, at which time many rural families slaughter the family hog and enjoy higher rates of meat consumption than at other times during the year. Given the low rate of meat consumption we observe among our sample infants, however, it is unclear if this same trend applies to the infant population used in this study. Overall, then, while we do not have any hard data on this question, we suspect that seasonal bias is not a significant issue, and if anything, the Hb levels we report in this study should be considered an upper bound, since our data was collected in the spring (April).

Another possibility we considered was whether other health conditions might be causing the high rates of anemia we observed in our study areas, such as thalassemia or hookworm. As it turns out, however, thalassemia in China typically occurs only in areas of China south of the Yangtze River, not in Shaanxi Province, where our study was conducted. Indeed, a search of hospital records in two of the largest teaching hospitals in Shaanxi Province and of the scientific literature on China’s northwestern provinces indicate no cases or suspected cases of thalassemia major in the past 30 years. Regarding hookworm, data from the National Survey of Human Parasites in China indicates a national average hookworm prevalence of 6.12%, with rates highest in the humid, tropical regions of southern China and lowest in the dry regions of northwest China, where our study was conducted [59]. While data on hookworm prevalence was not published separately by province, it is safe to assume that hookworm prevalence in the survey areas is well below 6% overall, and likely even lower for children in our age group who are not independently mobile. It is therefore unlikely that either of these two diseases is behind the anemia prevalence we report in this study.

Another possible explanation for the high anemia prevalence we observe might be malaria; however, since malaria is not prevalent in the sample area [60], we suspect that other factors, such as dietary insufficiencies, are more likely to be the root cause of the high anemia prevalence we report here.

One limitation of the study is its cross-sectional design, which does not allow us to identify causal relationships. Moreover, we were unable to conduct whole blood testing for nutritional deficiencies, and are therefore limited to considering hemoglobin as our sole indicator of micronutrient deficiency. Finally, since our data on infant feeding practices is based on caregiver recall, we cannot rule out the possibility of recall bias.

Another possible source of bias stems from the fact that our study children were identified based on a list of registered infants provided by the village family planning official, thus systematically excluding all unregistered children. The number of unregistered children has drastically declined in recent years, due to a combination of naturally declining fertility rates and loosening government policies. Indeed, a 2010 survey found the rate of unregistered children to be only around 0.12% [61]. We therefore believe that this bias is negligible.

## 5. Conclusions

Anemia is still a severe public health problem among 6 to 11 month old infants in rural China. Infants breastfed over 6 months of age had a higher risk of anemia. Infants who had ever been formula-fed had lower risk of anemia. Iron supplementation or home fortification while breastfeeding is recommended.
